# Early Functional Outcome of Resection and Endoprosthesis Replacement for Primary Tumor around the Knee

**DOI:** 10.5704/MOJ.1303.013

**Published:** 2013-03

**Authors:** AR Sharil, AH Nawaz, MZ Nor Azman, W Zulmi, WI Faisham

**Affiliations:** Department of Orthopaedics, Universiti Sains Malaysia, Kubang Kerian, Malaysia; Department of Orthopaedics, Universiti Sains Malaysia, Kubang Kerian, Malaysia; Department of Orthopaedics, Universiti Sains Malaysia, Kubang Kerian, Malaysia; Department of Orthopaedics, Universiti Sains Malaysia, Kubang Kerian, Malaysia; Department of Orthopaedics, Universiti Sains Malaysia, Kubang Kerian, Malaysia

## Abstract

We evaluated functional outcomes for patients who underwent surgery for resection and endoprosthesis replacement for primary tumours around the knee. We used the Musculoskeletal Tumour Society Scoring System (MSTS) for functional evaluations to compare differences between distal femur (DF) and proximal tibia (PT) placements. The study sample included 34 cases of distal femur and 20 cases of proximal tibia endoprosthesis replacement. Primary tumours were classified as follows: 33 osteosarcoma, 20 stage III giant cell tumour (GCT) and one case of mesenchymal chondrosarcoma. The mean MSTS score for both DF and PT endoprosthesis together was 21.13 (70.43%), and The MSTS scores for DF was 21.94 (73.13%) and PT was 19.75 (65.83%) Infection developed in 7 cases and 5 of which were PT endoprosthesis cases. Three deep infections required early, two-stage revision and resulted in poor MSTS scores. We conclude that endoprosthesis replacement for primary bone tumours had early good to excellent functional outcome. There were no differences in functional outcomes when comparing distal femur endoprostheses with proximal tibia endoprostheses.

**Key Words:**

functional outcome, bone tumour, knee, and endoprosthesis

## Introduction

The knee is the most frequent site of primary bone tumour^1^. A majority of the tumours arising in the knee can be treated with limb sparing surgery and result in good early and late functional outcomes^1^,^2^,^3^,^4^,^5^. Reconstruction methods include osteoarticular allografts, allograft prosthetic composites and segmental endoprosthesis replacement. Prosthesis placement allows immediate weight bearing, maintenance of joint mobility, and early return to activities of daily living^2^,^3^,^4^

Distal femur endoprosthesis replacement produces good early and late functional result compared to proximal tibia endoprostheses. The aim of this study was to evaluate and compare functional outcomes for prosthesis replacement, particularly the differences between use of the distal femur vs. proximal tibia.

## Materials and Methods

This study was a cross sectional evaluation of follow-up of 54 orthopaedic oncology patients (37 male, 17 female; mean age 26y, range 15-55y) seen between October 2007 and February 2008. Thirty-three patients had osteosarcoma, 20 patients had stage III giant cell tumour and one patient had mesenchymal chondrosarcoma. There were 34 distal femur and 20 proximal tibia endoprosthesis placements. We used Kinematic rotating-hinge prostheses on 44 patients (Stryker Howmedica Osteonic, Inc, Rutherford NJ) and a Finn rotating-hinge prostheses (Biomet Orthopedic, Inc., Warsaw, IN) on 10 patients. At the time of review, 9 osteosarcoma 3 patients with giant cell tumours survived with pulmonary metastases. The overall mean interval from actual surgery to review was 36.8 months (range, 10- 89 months), while the mean interval for the distal femur group was 39 months compared to 33 months for the proximal tibia group (not a statistically significant difference (p<0.05)).

Functional outcomes were evaluated using the musculoskeletal tumour society system^6^ (MSTS) by the first author through interview. (Table I) Further assessment of range of knee motion included goniometer measurements and the radiographs to check for early endoprosthesis failure using standard anteroposterior view and lateral views. Wound infection is defined based on modification of CDC criteria of surgical wound infection (1992)^7^.

The ethics committee of the Hasan Sadikin Hospital approved the study protocol and the study was conducted in accordance with the Helsinki Declaration (1975).

Data were analyzed using the SPSS software, version 12.0 for windows.

## Results

**Fig 1:**
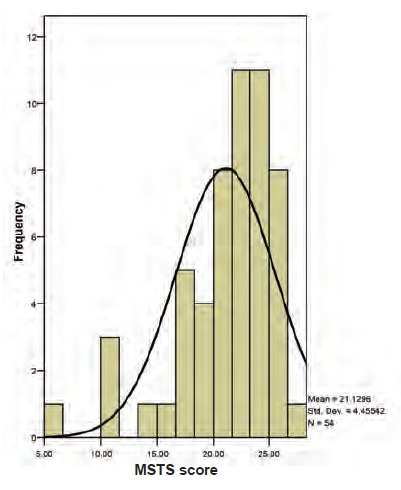
: Histogram showing overall Musculoskeletal Tumour Society (MSTS) score for both proximal tibia and distal femur endoprosthesis.

**Fig 2:**
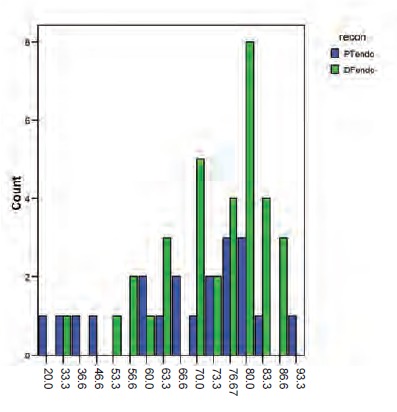
: Histogram showing distribution of Musculoskeletal Tumour Society Scoring System (MSTS) scores (percentage) for proximal tibia and distal femur endoprosthesis groups.

Functional outcomes, as measured by MSTS functional assessment were good to excellent in a majority of study patients (mean, 21.1 (70.43%); range, 6-28) (Figure 1). Patients in the distal femur group had a mean MSTS score of 21.9 (73.1%) compared to the proximal tibia group with a mean of 19.8 (65.8%). This difference between groups was not significant (Pearson chi-square value, 17.78; p=0.274) (Figure 2)

**Fig 3:**
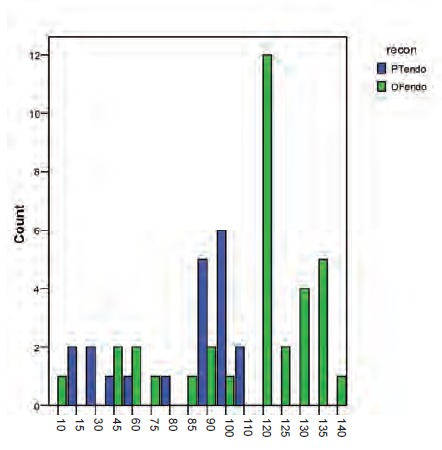
: Distribution of range of motion of knee joint of both types of reconstructions. Abbreviations: ROM, range of motion; PT endo, proximal tibia endoprosthesis; DF, distal femur endoprosthesis.

Detailed MSTS analysis (over 5 point assessments, (Table II)) revealed that for the pain component, the proximal tibia group had a mean of 3.9 compared to 4.2 for distal femur group, not a significant difference (Pearson Chi-square value, 2.91; p-0. 406). The mean functional score for the distal femur group was 3.3 compared to 3 in the proximal tibia group, also not a statistically significant difference. The mean emotional component score for the distal femur group was higher 3.2 vs. 2.8 for proximal tibia group, not significantly different). Also according to MSTS scoring, patients in the distal femur patient needed less support (mean score, 4.2 vs. 3.9 for proximal tibia group). A majority of the patients were ambulating without support at the time of the interview (11/20 in proximal tibia and 23/34 in distal femur) but one patient with proximal tibia endoprosthesis suffered complications of infection that resulted in the need for crutches to walk. Most patients were able to walk slightly limited distance (grade 4, 11/20 patients in proximal tibia group vs. 21/34 patients in distal femur group). The mean score of walking component was 3.4 in proximal tibia compared to 3.6 in distal femur with no statistical different (Figure 3)

The mean degree of knee flexion for DF endoprosthesis patients was 108.38º compared to 77.25º in the PT group. Knee flexion in the DF group was significantly better compared to the PF group (Pearson chi-square, 38.481; p- 0.001) (Figure 3)

Infection developed in 7 patients (5 in the proximal tibia group and 2 in the distal femur group. Four of the infections were superficial and eradicated with prolonged antibiotics and local dressing. MSTS scores for those who had superficial infection were good with a mean score of 18.3. Three patients (2 from the proximal tibia group and 1 from the distal femur group) required early debridement, removal of implant and use of a cement spacer followed by secondary surgery. Their mean score was 10.3 including one patient from the proximal tibia group who had a score of 6 due to the need for multiple debridement procedures and resultant chronic persistent infection.

One patient in the distal femur endoprosthesis group required early revision surgery due to mechanical failure. He had segment disintegration and fracture and underwent removal of the original endoprosthesis and insertion of a new component. His final outcome was good with a MSTS score of 23. Another patient with proximal tibia giant cell tumour had local recurrence 2 years following the initial procedure. Marginal resection and a free latissimus dorsi flap cover followed by local radiotherapy controlled the disease locally. The patient was treated with radiation therapy because of positive microscopic margins and contamination over the surgical margins. The patient remained with a flexion contracture of 2°, was able to ambulate with walking stick and had a MSTS score of 15.

## Discussion

The knee is the most common site for primary bone tumours and a majority are in the distal femur^1^,^2^,^3^,^4^,^5^,^8^,^9^. Osteosarcoma is the most common type of malignant bone tumour in the region of the knee^1^,^2^,^3^,^4^,^5^,^8^. Stage III giant cell tumour (GCT) of the bone is a locally aggressive disease requiring wide resection for local control; a majority of our giant cell tumour patients presented with aggressive stage III GCT^9^. Limb salvage surgery is an accepted of treatment for tumours around the knee. Amputation should be reserved for tumours with multi-compartment soft tissue and major neurovascular involvement and a high risk of local recurrence. Megaprosthesis is now the method of choice to restore function and results in optimal patient satisfaction^1^,^2^,^3^,^4^,^5^,^8^.

Endoprosthesis reconstruction for distal femur tumours enable immediate weight bearing, maintenance of joint mobility, and early return to activities. Functional outcomes were generally good to excellent with good range of knee motion for activities of daily living (median flexion of 110°)^1^,^3^,^4^,^5^,^10^. Improvements in mechanical rotating-hinge design by enhanced dispersion of joint stress during motion has decreased endoprosthesis loosening rates. The long-term survival rate has been reported as 90% at 5 years and 80% at 10 years and the knee function is good for long periods^1^,^2^,^3^,^4^,^5^,^10^,^11^. Distal femur and proximal humerus endoprostheses have better overall long-term survival rates, followed by the proximal femur, proximal tibia and distal humerus in tumour reconstruction^1^,^2^.

The proximal tibia region is more difficult for wide resection and subsequent reconstruction, due to the close proximity of the tumour to major neurovascular bundles and inadequate soft tissue coverage. Reconstruction of the extensor mechanism is a weakness in reconstructions involving proximal tibia resection; in fact, failure is common for this procedure^12^,^13^,^14^,^15^. Proximal tibia replacement results in poorer functional outcome compared to distal femur replacements. The risk of infection and early re-operation is higher and final survival of prosthesis is shorter for proximal tibia replacements, and rates for secondary rupture of the extensor mechanism range from 4%-15%^12^,^13^. The use of a medial gastrocnemius flap dramatically lowers the infection rate and improves resultant knee extension^14^, but the outcome is still poorer compare to distal femur procedures, as the patella tendon re-attachment is not biological and there is a tendency to slip, avulsion it causes extension lag. Furthermore, late rehabilitation and extensive surgical procedures associated with the flap lead to fibrosis that limits knee range of motion. We used a medial gastrocnemius flap as part of extensor mechanism reconstruction and repaired soft tissue in 90° flexion for all proximal tibia reconstruction patients. All patients were braced in full extension for 6 weeks before starting knee range of motion rehabilitation. (Figures 5 and 6)

Our study revealed that, although the range of knee motion was restricted in the proximal tibia group the difference was not was not statistically significant. The overall short-term functional outcomes were also comparable for each measure in the MSTS evaluation. Prosthesis-related events generally follow predictable patterns as wound related complications and recurrence occur early in the postoperative period 1,2,3,4,5,11. Superficial infection did not contribute to poor functional outcome but deep infection required removal of implant in one patient and resulted in a poor functional outcome. Four patients with superficial infection, controlled with prolonged antibiotics, had comparable outcomes to the patients without infection. Three patients had deep infection that required repeated debridement, spacers and re-implantation; as would be expected, they had poor functional outcomes with a mean score 10.3 including one patient with a score of 6. These finding are similar to those of Henderson et al. who noted that infection was the most important cause of early failure and that patients with proximal tibia replacement had higher failure rates than those with distal femur procedures^16^. Infection rates are reported to be higher in proximal tibia procedures compared to others. The superficial location of proximal tibia prostheses and its precarious blood supply might be the main causes of this problem.

**Table I:**
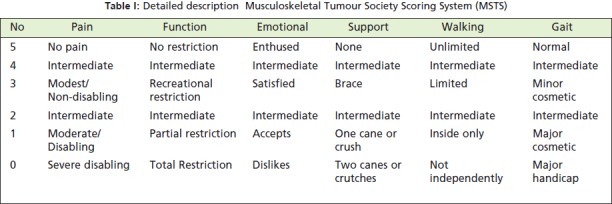
: Detailed description Musculoskeletal Tumour Society Scoring System (MSTS)

**Table II:**
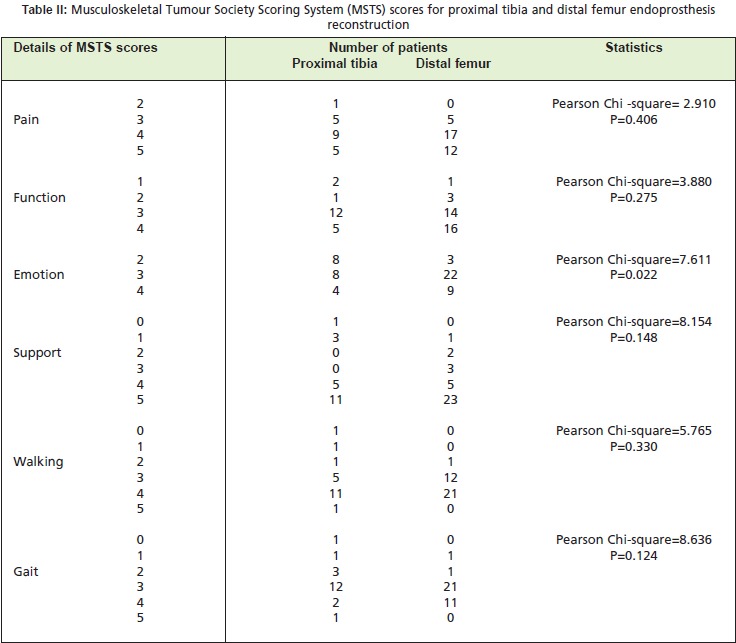
: Musculoskeletal Tumour Society Scoring System (MSTS) scores for proximal tibia and distal femur endoprosthesis reconstruction

**Fig 4:**
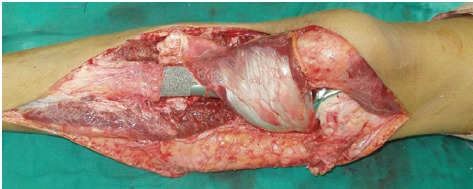
: Photograph showing proximal tibia endoprosthesis reconstruction with medial gastrocnemius flap.

**Fig 5:**
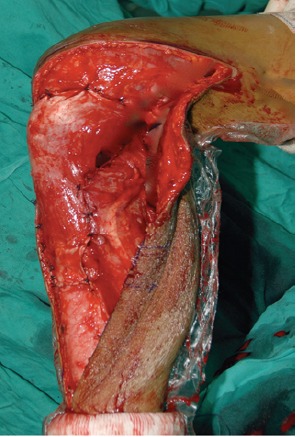
: Photograph of reconstruction of medial gastrocnemius flap and extensor mechanism showing knee at in 90° of flexion.

## Conclusion

Overall early functional outcomes of resection and endoprostheses placement of the distal femur and proximal tibia tumour are good. There is no difference in functional outcome between both anatomical sites.
